# Association between visual acuity, lesion activity markers and retreatment decisions in neovascular age-related macular degeneration

**DOI:** 10.1038/s41433-020-0799-y

**Published:** 2020-02-17

**Authors:** Usha Chakravarthy, Natasha Pillai, Annie Syntosi, Lorna Barclay, Catherine Best, Alexandros Sagkriotis

**Affiliations:** 1 Centre for Experimental Medicine, Institute of Clinical Science, Queen’s University Belfast, Belfast, UK; 2 IQVIA, Basel, Switzerland; 3 Novartis Pharma AG, Basel, Switzerland

**Keywords:** Outcomes research, Macular degeneration

## Abstract

**Background/objectives:**

To investigate the association between optical coherence tomography (OCT) markers of lesion activity and changes in visual acuity (VA) during anti-vascular endothelial growth factor (anti-VEGF) therapy of eyes diagnosed with neovascular age-related macular degeneration (nAMD); and how VA and OCT markers are considered in physicians’ decision to retreat with anti-VEGFs.

**Subjects/methods:**

Retrospective, non-comparative, non-randomised cohort study involving electronic medical record data collected from 1190 patient eyes with nAMD diagnosis at two sites in the United Kingdom. Two sub-cohorts consisting of 321 and 301 eyes, respectively, were selected for analyses.

**Results:**

In 321 eyes, absence of IRF or SRF at ≥2 clinic visits resulted in a gain of five ETDRS letters from baseline, compared with two letters gained in eyes with <2 clinic visits with absence of IRF (*p* = 0.006) or SRF (*p* = 0.042). Anti-VEGF treatment was administered at 421 clinic visits, and 308 visits were without treatment. Comparing treatment visits with non-treatment visits, the maximum difference in frequency of OCT markers of lesion activity were for intraretinal fluid (IRF; 24% versus 5%) and subretinal fluid (SRF; 32% versus 5%). Pigment epithelial detachment (PED) was reported in 58% of treatment visits compared with 36% in non-treatment visits. VA loss was not a consistent trigger for retreatment as it was present in 63% of injection visits and in 49% of non-injection visits.

**Conclusions:**

Retreatment decision making is most strongly influenced by the presence of IRF and SRF and less by the presence of PED or VA loss.

## Introduction

Neovascular age-related macular degeneration (nAMD) is a late-stage manifestation of a chronic degenerative disease affecting the macular retina [1, 2]. In nAMD, anomalous pathologic blood vessels arising from the choroid disrupt the anatomy and function of the neurosensory retina, a process that is largely driven by the pro-angiogenic cytokine vascular endothelial growth factor (VEGF) [3, 4]. Anti-VEGF therapy for the treatment of nAMD with ranibizumab was introduced following the pivotal ANCHOR and MARINA studies [4, 5] and with aflibercept following the VIEW 1 and VIEW 2 studies [6]. Although initially evaluated using monthly dosing regimens, subsequent clinical studies suggested that after a loading phase of three monthly injections, *pro re nata* (PRN) anti-VEGF treatment is also effective [7]. The HARBOR study further demonstrated that an optical coherence tomography (OCT)-guided PRN dosing strategy seems to be as beneficial as a fixed monthly dosing regimen [8]. Indeed, using anatomical changes detected by OCT to guide patient-specific PRN dosing can improve and maintain visual acuity (VA) with fewer injections, although monthly monitoring visits are still required [9]. Other treatment regimens, such as OCT-guided treat-and-extend dosing, have also been evaluated [10, 11].

Despite the evidence for the benefits of anti-VEGF therapy in clinical trials, a recent real-world observational study in the UK showed that the incidence of new blindness due to nAMD increased from year 1 (5.1%) to year 4 (15.6%) in anti-VEGF-treated eyes [12]. However, the reasons underpinning this VA loss remain obscure. A number of small studies have explored potential explanations for this decline in VA by examining anatomic indicators of disease activity detected on OCT and have identified subretinal fluid (SRF) and intraretinal fluid (IRF) at baseline as possible biomarkers for better and worse treatment outcomes, respectively [13–18]. In line with these observations, nAMD guidelines from key professional bodies recommend using OCT-based criteria as markers of lesion activity [8, 19–21].

However, in real-world clinical practice, the impact of OCT markers of lesion activity and VA change on physicians’ retreatment decisions with anti-VEGF is poorly understood [22]. Real-world evidence (RWE) studies show variability of functional outcomes over time and suggest that clinicians may not use these markers of lesion activity and VA change consistently to guide retreatment [23, 24]. Clinicians’ decisions to treat with anti-VEGFs may also be influenced by other factors such as a high treatment burden for patients, caregivers and healthcare providers [25].

The present study was designed to explore the association between OCT markers of lesion activity measured after the anti-VEGF loading period and VA, measured after 1 year of anti-VEGF treatment. Whether the sustained presence or absence of retinal fluid (IRF and SRF) impacts visual outcome was of particular interest. A further aim was to identify the main drivers for clinicians’ retreatment decisions. In this report we describe the associations between OCT markers of lesion activity and VA outcomes during the maintenance phase and how both parameters are used in routine clinical practice for the treatment of nAMD patients.

## Subjects and methods

### Objectives

The primary objective of this RWE study was to investigate the association between OCT markers of lesion activity during the anti-VEGF maintenance phase (months 3–12) and change in VA from baseline at month 12. The secondary objective was to investigate how physician recorded measures of VA and OCT markers of lesion activity influenced their decisions to treat during the maintenance phase of the first year of anti-VEGF treatment.

### Study design and data sources

**Fig. 1:**
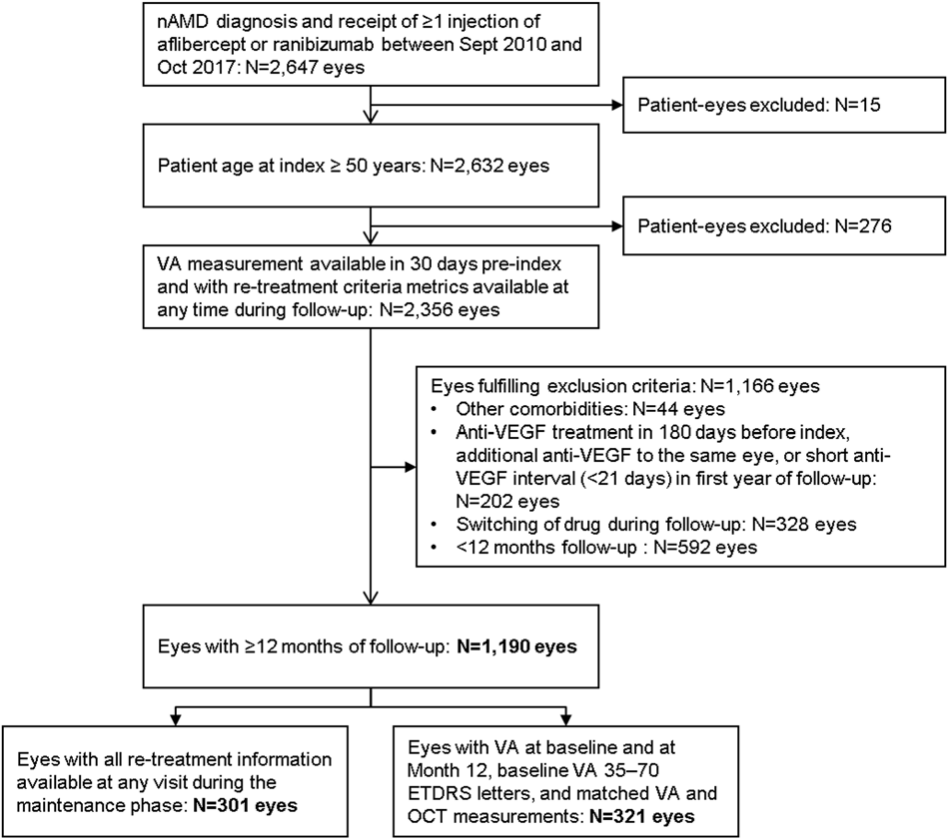
Selection workflow, patient disposition and definition of the study cohort. ETDRS Early Treatment Diabetic Retinopathy Study, nAMD neovascular age-related macular degeneration, OCT optical coherence tomography, VA visual acuity, VEGF vascular endothelial growth factor.

This was a retrospective, non-comparative, non-randomised cohort study of the characteristics of nAMD patients, conducted using data from Medisoft electronic medical records (EMR) collected in a standardised manner at two sites in the United Kingdom. In order to investigate the primary and secondary objectives, two sub-cohorts were derived from the main cohort. The primary objective was investigated in a sub-cohort consisting of eyes with VA at baseline and at month 12 and a baseline VA between 35 and 70 Early Treatment Diabetic Retinopathy Study (ETDRS) letters (the ‘OCT markers of lesion activity cohort’). The secondary objective was investigated in a sub-cohort made up of eyes with all retreatment information available at any visit during the anti-VEGF maintenance phase (i.e. months 3–12; the ‘retreatment criteria cohort’; Fig. 1).

### Participants

The dataset was scrutinised to identify patients aged ≥50 years with a diagnosis of nAMD only that had received at least one licensed anti-VEGF injection between 1st September 2010 and 4th October 2017 without a treatment switch during the maintenance phase (high level eligibility criteria). The index date was defined as the date of the first anti-VEGF injection. The eye was the unit of analysis; therefore, a patient may have one or two study eyes.

### Outcome measures

The Medisoft EMR system offers users a dropdown ‘retreatment criteria’ list including indicators (measures) of VA loss and OCT markers of lesion activity that can be simultaneously selected when present. Dropdown fields dealing with ‘retreatment criteria’ were: a loss of >5 ETDRS letters compared with a patient’s highest VA; loss of >5 ETDRS letters since the previous clinic visit; (i.e. measures of VA loss); presence of the following: SRF; diffuse IRF; intraretinal cysts (IRC); pigment epithelial detachment (PED); macular haemorrhage (MH); exudate; or a central retinal thickness (CRT) increase ≥20% since the previous clinic visit (i.e. OCT markers of lesion activity).

### Statistical analysis

Associations between change in VA during the maintenance phase of anti-VEGF treatment (months 3–12) and markers of lesion activity were assessed. Measures of VA loss obtained by extraction from the dropdown menu were cross checked and validated using the EMR records of VA measurements at available visits. VA measures and OCT markers of lesion activity were classified as relating to injection or non-injection clinic visits. A 21-day window was allowed to pair records of OCT markers of lesion activity with VA measurements. A time window of 30 days was applied to assign a clinic visit to baseline and to months 3–9, and a 60-day window was applied for month 12.

Descriptive statistics were tabulated for demographic and clinical characteristics and outcome variables. Continuous variables were summarised as number of observations, means, standard deviations (SD) and medians (interquartile range; IQR). Categorical variables were summarised as counts and proportions, with missing data considered a separate category. VA and OCT markers of lesion activity during injection and non-injection visits were presented as a proportion of visits, where the OCT or VA feature was recorded.

The number of patient eyes with a record of VA and OCT markers of lesion activity at different time points was tabulated. Each OCT marker of lesion activity was analysed separately. Patient eyes were grouped by the number of visits at which fluid was absent (i.e. 0, 1, 2, 3, 4, 5 or ≥6 visits). Eyes were also dichotomised by presence of fluid in <2 or ≥2 visits during the maintenance phase. The non-parametric Mann–Whitney *U* test was used to identify associations between functional outcomes (VA) and OCT markers of lesion activity (IRF, SRF). To examine associations between change in VA from baseline to month 12 and the number of visits with absence of IRF or SRF, a locally weighted regression model with a 95% confidence interval was fitted to the data. In the derivation of inferential statistics, VA change from baseline was the dependent variable and number of visits without IRF and/or SRF dichotomised to <2 or ≥2 visits during the maintenance phase (months 3–12) was the independent variable.

### Ethics statement

This study was designed, conducted, and reported in accordance with the guidelines for Good Pharmacoepidemiology Practices of the International Society for Pharmacoepidemiology, the Strengthening the Reporting of Observational Studies in Epidemiology guidelines and the ethical principles stated in the Declaration of Helsinki [26–28].

## Results

### Patient disposition

Two clinical sites in the UK contributed a total of 2647 study eyes. The first anti-VEGF injection was given between 1st September 2010 and 4th October 2017 (index period) and patients were followed for a minimum of 12 months. Two thirds (67%) of patient eyes were eligible for inclusion after applying the high level exclusion criteria. On restricting the analysis to eyes with a minimum of 12 months of follow-up, 1190 (45%) eyes (i.e. the main cohort) remained evaluable. The sub-cohort in which OCT markers of lesion activity was investigated was made up of 321 eyes (12.1%) and the retreatment criteria sub-cohort consisted of 301 eyes (11.4%) (Fig. 1). Supplementary Table 1 provides the number of patient eyes at each time point with all available OCT markers of lesion activity. VA and OCT measurements were collected on the same day in >99.5% of all included study eyes.

### Baseline characteristics

Baseline characteristics by high level eligibility criteria and for the sub-cohorts selected for analyses were similar for age, gender and baseline VA (Table 1). The majority (63.1%) of patients had their index anti-VEGF injection between 2014 and 2017, and 88% of patients received unilateral anti-VEGF treatment (Table 1). A higher proportion of patients had OCT images in the second half of the study period (2014–2017) compared with those in the early period (2010–2013).

**Table 1:** Baseline characteristics at the time of the index injection.

	Main cohort (*N* = 1190 eyes from 1065 patients)	OCT markers of lesion activity sub-cohort (*N* = 321 eyes from 307 patients)	Retreatment criteria sub-cohort (*N* = 301 eyes from 279 patients)
Age at index (patient)			
Mean years (SD)	79.1 (6.3)	79.5 (6.4)	80.2 (6.5)
Gender (patient): (*n*, %)			
Male	404 (37.9%)	119 (38.8%)	113 (40.5%)
Female	661 (62.1%)	188 (61.2%)	166 (59.5%)
Year of index injection (eye): (*n*, %)			
2010	49 (4.1%)	3 (0.9%)	0 (0.0%)
2011	121 (10.2%)	6 (1.9%)	0 (0.0%)
2012	130 (10.9%)	12 (3.7%)	0 (0.0%)
2013	138 (11.6%)	19 (5.9%)	0 (0.0%)
2014	206 (17.3%)	72 (22.4%)	8 (2.7%)
2015	205 (17.2%)	75 (23.4%)	106 (35.2%)
2016	192 (16.1%)	77 (24.0%)	108 (35.9%)
2017	149 (12.5%)	57 (17.8%)	79 (26.2%)
Eyes treated (patient): (*n*, %)			
Unilateral	940 (88.3%)	293 (95.4%)	257 (92.1%)
Bilateral	125 (11.7%)	14 (4.6%)	22 (7.9%)
Eyes treated (patient): (*n*, %)			
Left	431 (40.5%)	120 (39.1%)	126 (45.2%)
Right	509 (47.8%)	173 (56.4%)	131 (47.0%)
Both	125 (11.7%)	14 (4.6%)	22 (7.9%)
VA study eye ETDRS letters^a^ (eye)			
Mean (SD)	50.8 (15.3)	54.7 (8.7)	51.0 (15.3)

*ETDRS* Early Treatment Diabetic Retinopathy Study, *OCT* optical coherence tomography, *SD* standard deviation.

^a^VA at index or in the 30-day period before the index injection.

### Association of OCT markers of lesion activity with VA

**Fig. 2:**
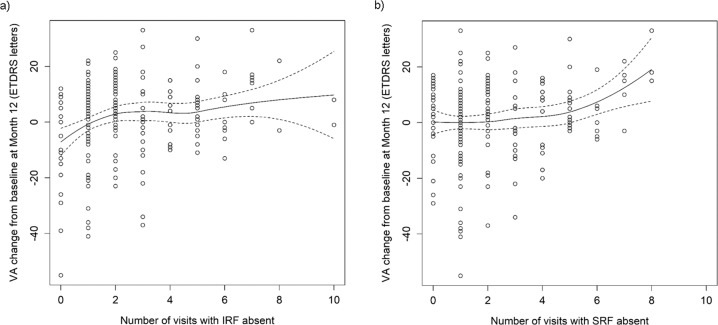
Association between absence of IRF and SRF and change in VA from baseline to month 12. Graphical representation of the association between number of clinic visits (*N* = 321 eyes) with absence of either IRF (**a**) or SRF (**b**) and VA change from baseline at the end of the first year of treatment with anti-VEGF therapies (month 12). ETDRS Early Treatment Diabetic Retinopathy Study, IRF intraretinal fluid, SRF subretinal fluid, VA visual acuity.

A positive correlation was observed between the number of visits during the maintenance phase with a record of absence of fluid (IRF and SRF) and gain in VA (Fig. 2a, b). Data suggest that the more monitoring visits associated with absence of fluid the better the VA gains at the end of year 1 (Supplementary Table 2A, B). After dichotomising data pertaining to visits to <2 versus ≥2 to allow for equal number of eyes within each cluster, the differences identified were statistically significant (IRF: three ETDRS letters difference between the groups, *p* = 0.006; SRF: three ETDRS letters difference between the groups, *p* = 0.042). Known poor predictors of VA at the end of year 1, such as increased age, absence of loading phase, undertreatment and high baseline VA were balanced across all clusters (Supplementary Table 2C).

### Sensitivity analysis

Due to the real-world origin of the study cohort, reporting of OCT markers of lesion activity during the maintenance period (months 3–12) was missing from a large subset of eyes (113/321 eyes) (Supplementary Table 3). In the sensitivity analyses performed, non-injection visits were assumed to be a proxy for absence of IRF and SRF, while clinic visits associated with injections a proxy of presence of IRF and SRF. Most of the missing OCT markers of lesion activity came from the non-injection visits (73%). Sensitivity analyses after substitution of missing data points confirmed the association of VA with IRF (*p* = 0.036), but not with SRF (*p* = 0.111; Supplementary Table 3).

### Association of retreatment decisions with markers of lesion activity

**Fig. 3:**
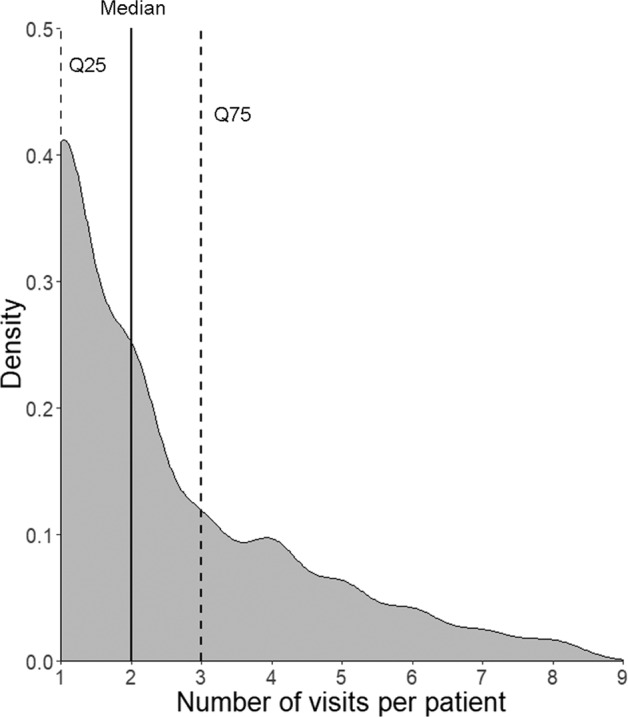
Density plot showing the distribution of number of visits per patient eye (*N* = 301 eyes, 729 visits) during the study maintenance phase (i.e. months 3–12). Q25 25% quartile, Q75 75% quartile. Dotted lines, 25% and 75% quartiles, respectively; solid line: median.

With respect to retreatment decisions, the 301 evaluable patient eyes contributed 729 clinic visits in total during the maintenance phase (months 3–12). The median (IQR) number of visits was 2.0 (IQR: 1.0–3.0) (Fig. 3). Of these 729 visits, anti-VEGF treatment was administered at 421 clinic visits (i.e. injection visits), whereas treatment was not administered at the other 308 clinic visits (i.e. non-injection visits).

**Fig. 4:**
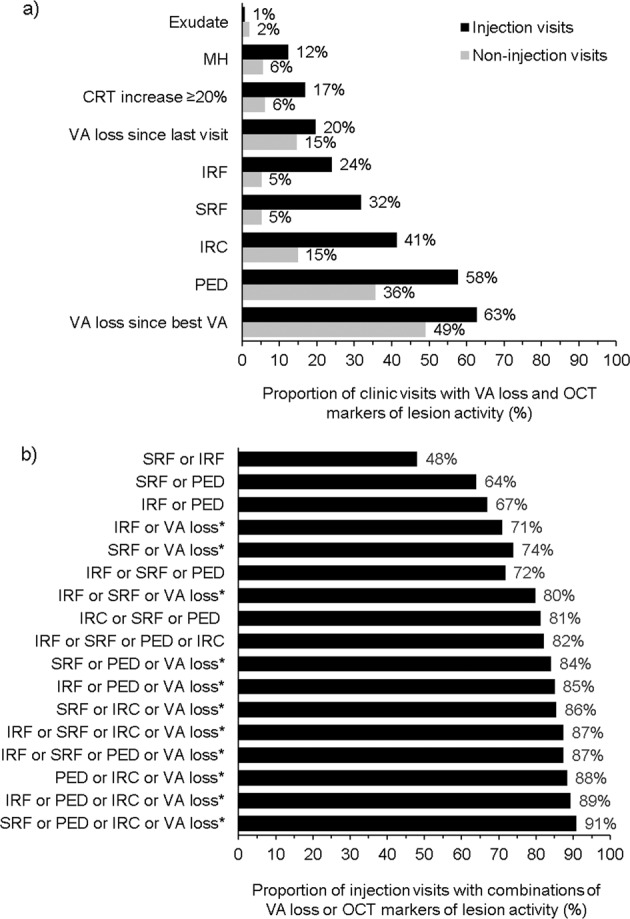
Bar graph showing proportions of visits with OCT markers of lesion activity and VA loss since last visit or since best VA at injection (black bars; *N* = 421) and non-injection (grey bars; *N* = 308) clinic visits in order of frequency. Exudate was the least frequently recorded OCT marker of lesion activity, accounting for fewer than 2% of both injection and non-injection visits. VA loss of >5 ETDRS letters since best VA was most frequent at injection visits (panel **a**). The occurence of combinations of these features observed at injection visits is presented in panel **b**. The asterisk symbol indicates the VA loss of >5 ETDRS letters since best VA. CRT central retinal thickness, IRC intraretinal cyst, IRF intraretinal fluid, MH macular haemorrhage, OCT optical coherence tomography, PED pigment epithelial detachment, SRF subretinal fluid, VA visual acuity.

Figure 4a shows the frequency at which OCT markers of lesion activity and/or VA loss of >5 ETDRS letters since the preceding visit or from best measured VA were present at injection and non-injection visits. Concordance of OCT markers of lesion activity and VA losses at injection visits are shown in Fig. 4b.

All markers indicating lesion activity, individually (with the exception of exudate) or in various combinations, were more frequently reported at injection visits compared with non-injection visits. Exudate, CRT increase ≥20%, IRC, IRF, MH, PED, SRF, and loss of >5 ETDRS letters since the previous visit were absent at >85% of all non-injection visits (Fig. 4a). On examining the relative prevalence of the features of interest at injection versus non-injection visits, the prevalence of SRF was found to be ~sixfold higher (32% versus 5%), IRF fivefold higher (24% versus 5%) and PED 1.5-fold higher (58% versus 36%) at injection visits than at non-injection visits. The lowest differences between injection and non-injection visits were in the prevalence of loss of >5 ETDRS letters since either the previous visit or since best VA (Fig. 4a).

The proportion of clinic visits with an anti-VEGF injection increased with the number of markers present (one marker: 24–63%; two: 48–81%; three: 72–88%; four: 87–91%). Notably, the presence of IRF, SRF or VA loss of >5 ETDRS letters compared with a patient’s best VA was associated with 80% of injection visits (Fig. 4b).

At injection visits with a record of both VA and OCT biomarkers of lesion activity (*N* = 336 injection visits), VA loss since the best VA was the most prevalent marker reported, either alone (40.0% of visits), or in combination with IRF and/or SRF (38.7%). SRF without VA loss was only reported at 11.3% of injection visits; IRF without VA loss in 7.4% of visits and IRF plus SRF without VA loss at 2.7% of injection visits (Supplementary Fig. 1).

### Validation of data obtained from the EMR dropdown menu versus actual VA record on the EMR system for the retreatment decision analysis

The concordance between VA measurements directly extracted from the Medisoft EMR system and the VA information recorded in the retreatment criteria dropdown lists was tested. For eyes with VA loss of >5 ETDRS letters since the previous visit, 92% were concordant with the VA recorded in the EMR. For eyes with VA loss >5 ETDRS letters since the best VA measurement, 61% of observations were concordant with a change in VA from best VA record to the selected visit.

## Discussion

We examined the association between OCT markers of lesion activity and visual outcome in patients with nAMD during the maintenance phase of anti-VEGF treatment during 12 months of follow-up. After adjustment for potential confounders (age, baseline VA, loading phase, and number of injections) which have been previously shown to impact VA outcomes at month 12 [14, 23], patient eyes with at least two visits with absence of IRF or SRF demonstrated significantly higher VA gains compared with eyes with less clinic visits with absence of fluid.

These findings are in agreement with previous studies reporting that the presence of IRF at baseline and during treatment is detrimental to vision [13, 17]. Furthermore, EURETINA guidelines highlight that baseline presence of IRF is a predictor of poor VA outcomes following anti-VEGF treatment [8]. Similarly, a recent, retrospective, 1-year follow-up study of anti-VEGF treated nAMD eyes reported that presence of IRF was associated with low VA at baseline and with no vision gains after 1 year of treatment [29].

An important objective of the present study was to understand determinants of physicians’ decisions to retreat or withhold treatment and how this was aligned with clinical guidelines. In this context we observed that a record of presence of retinal fluid (SRF, IRF) was at least fourfold more likely to be present at an injection visit compared with a non-injection visit, indicating that these two OCT markers of lesion activity are key drivers for physicians to order anti-VEGF retreatment. This means that physicians base their decision to inject not only on VA decline, but also on the presence of fluid, as suggested by many clinical guidelines [8, 19–21].

Due to the complex pathophysiology of the disease and the presence of multiple abnormalities that could justify a decision to inject, the current study was not limited to the presence of IRF or SRF, but also examined other OCT markers of lesion activity. One of those that did stand out was PED, which was a frequently reported OCT marker of lesion activity that was present both at injection and non-injection visits. Physicians appear to tolerate the presence of a PED in the absence of IRF or SRF without considering retreatment. Our data bears out the view that a residual PED without other accompanying signs of lesion activity is considered benign. The presence of a PED at non-injection visits is also consistent with EURETINA guidance that states that in the absence of concomitant IRF or SRF, the presence of PED can be managed through frequent monitoring without anti-VEGF treatment intervention [8]. Furthermore, an OCT angiography study of anti-VEGF treated eyes with type 1 neovascularization and PED suggests that stable PED may be beneficial when it is not associated with other markers of lesion activity, and cautions against anti-VEGF overtreatment of PED, which agrees well with our real-world observations of high PED prevalence at non-injection visits [30].

We observed that concordance with the change in VA since the last visit was close to 91%, but for eyes with VA loss >5 ETDRS letters since the best VA measurement, only 61% of observations were concordant with a change in VA from best VA record to the selected visit, which suggests that clinicians overestimate the VA loss compared to the best recorded VA in real-world clinical practice. We interpret this finding as indicating that physicians tend to use information that is closer to the actual visit rather than that seen during the loading phase when the highest gains in VA occur. Nonetheless, since this study aimed to describe physicians’ decision to treat, the information, as recorded in the Medisoft system, was used to address all related research questions.

With regard to our study design, we analysed data collected during the maintenance phase (months 3–12), as the current paradigm is to deliver treatment during the loading phase irrespective of the absence of OCT markers of lesion activity and also without reference to changes in VA during this period [31]. We also chose to restrict our analysis to eyes with a baseline VA between >35 and <70 ETDRS letters in order to better detect associations between anatomical and functional outcomes through avoiding ceiling and floor effects [25]. However, restricting our analysis to eyes in the middle range of VA may have introduced bias in terms of how patients with very good or very poor vision are managed.

Our study suffers from a number of limitations. On application of pre-specified criteria, data from only a subset of eyes could be included, and we therefore consider the analysis as a pilot study for hypothesis generation. Nonetheless, our attrition rates (~55%) are similar when compared with other cohorts of RWE studies published in the literature, which have reported losses of between 55 and 89% [32–34]. A larger RWE cohort study with quantification of retinal fluid volume and a longer follow-up period is planned to confirm the robustness of the present results. In sensitivity analyses of data from visits without injections, we observed findings that supported our main conclusions, but we assumed that injection free visits were a proxy for the absence of IRF or SRF. Other potential limitations of this real-world study are that we could only consider information available in the structured EMR (this data were not independently validated), the real-world study cohort was heterogeneous in nature, VA data recorded as logMAR was transformed to ETDRS letters, and presence or absence of fluid was reported by physicians without external validation.

Due to the real-world nature of this study and the manner of data collection in the EMR, it was difficult to account for the patterns of treatment (i.e. PRN or treat and extend). However, we speculate that for the majority of patients, a PRN regimen was used because the number of injection and non-injection visits should otherwise have been the same, and this was clearly not the case. Given that fixed time treatment is difficult to achieve in real life, due to comorbidities, low adherence, scheduling conflicts and other patient related factors we are not surprised.

In conclusion, this UK real-world study suggests that physicians generally treat IRF and SRF, as tolerance of IRF and SRF appears to be detrimental to the long term visual outcomes. We also confirmed the association between the absence of IRF or SRF at clinic visits and better VA outcomes in nAMD at the end of the first year of treatment with licensed anti-VEGFs.

## Summary

### What was known before

- Macular fluid and vision loss are criteria used for anti-VEGF retreatment decisions in eyes with nAMD. However, the extent to which vision loss and different types of macular fluid contribute to the real-world decision to retreat with anti-VEGF injections is poorly characterised.

### What this study adds

- Real-world anti-VEGF retreatment decision making is most strongly influenced by the presence of intraretinal and SRF and less by the presence of PED or vision loss.

## Supplementary information

Supplemental Figure 1

Supplemental Table 1

Supplemental Table 2

Supplemental Table 3
