# Supplementary material for: Association between visual acuity, lesion activity markers and retreatment decisions in neovascular age-related macular degeneration
Source: Eye (Lond). 2020 Feb 17;34(12):2249–56. doi: 10.1038/s41433-020-0799-y (PMC7784949; doi:10.1038/s41433-020-0799-y)

**Supplementary figure 1.** Prevalence of the retreatment criteria IRF, SRF, and VA loss >5 ETDRS letters since best VA at injection visits (N=336 injection visits with a record of OCT biomarkers of lesion activity). VA loss >5 ETDRS letters since last VA only was observed at 134/336 visits (40.0%); IRF only at 25/336 visits (7.4%); and SRF only at 38/336 visits (11.3%). VA loss was observed in combination with IRF and/or SRF at 130/336 injection visits (38.7%). At 9/336 visits (2.7%), SRF and IRF was detected without any VA loss. ETDRS, Early Treatment Diabetic Retinopathy Study; IRF, intraretinal fluid; OCT, optical coherence tomography; SRF, subretinal fluid; VA, visual acuity.


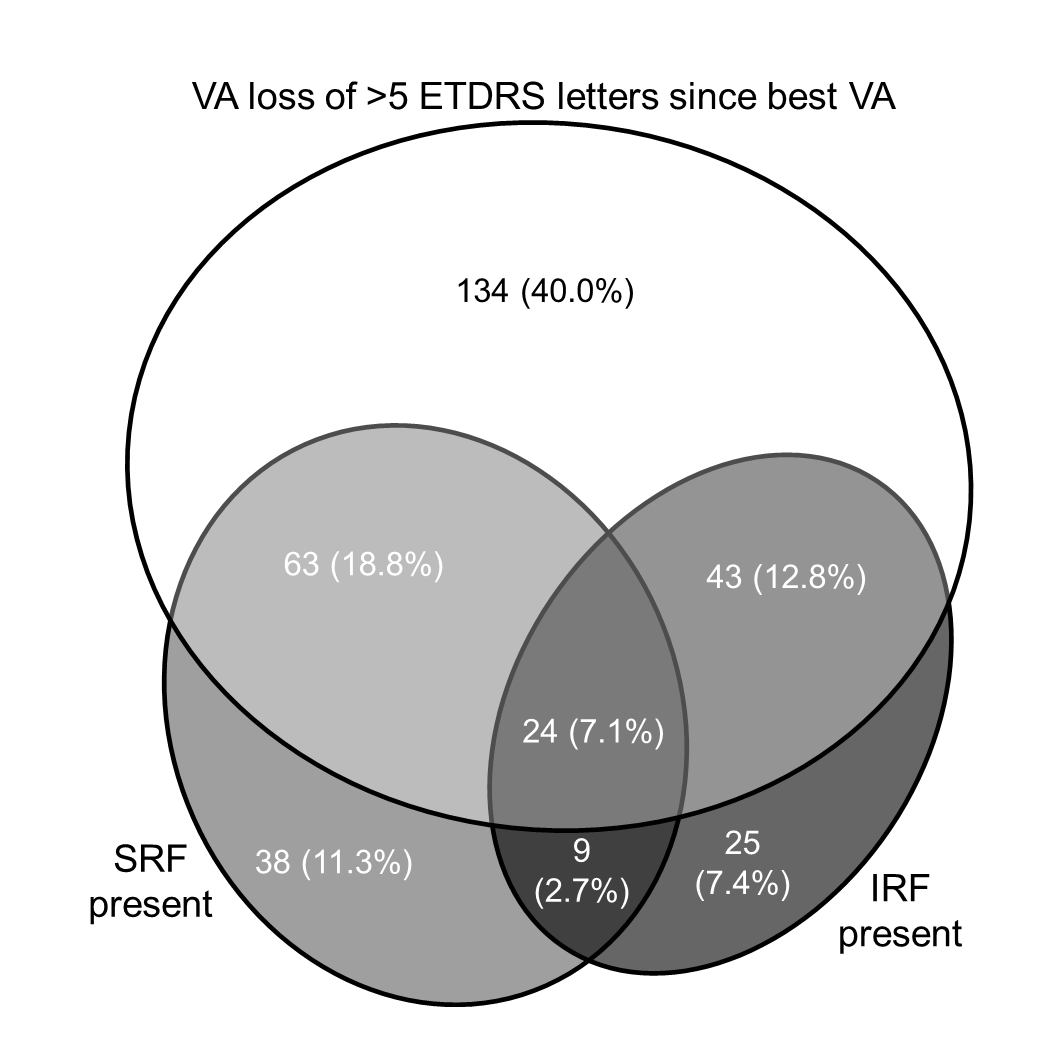

Supplement: Supplementary file 1 — Supplemental Figure 1 [file 41433_2020_799_MOESM1_ESM.docx]
