# Supplementary material for: Association between visual acuity, lesion activity markers and retreatment decisions in neovascular age-related macular degeneration
Source: Eye (Lond). 2020 Feb 17;34(12):2249–56. doi: 10.1038/s41433-020-0799-y (PMC7784949; doi:10.1038/s41433-020-0799-y)
Supplement: Supplementary file 2 — Supplemental Table 1 [file 41433_2020_799_MOESM2_ESM.docx]

**Supplementary table 1.** Study eye attrition due to missing OCT data in the EMR dataset. *The most recent VA and OCT measurement within a 30 day window was used to match measurements to clinic visits at Months 3–9, and a 60-day window was used at Month 12. **CRT increase ≥20%, exudate, MH, IRC, IRF, PED, SRF. IRC, intraretinal cyst; IRF, intraretinal fluid; MH, macular haemorrhage; PED, pigment epithelial detachment; SRF, subretinal fluid; VA, visual acuity.

| **Time*** | **Patient-eye counts with VA** | **Patient-eye counts with anatomical features**** | **Patient-eye counts with anatomical features** during the maintenance phase** |
| --- | --- | --- | --- |
| **Baseline** | 321 | 13 |  |
| **Month 3** | 305 | 56 | 51 |
| **Month 6** | 297 | 67 | 117 |
| **Month 9** | 287 | 84 | 66 |
| **Month 12** | 321 | 110 | 208 |
