# Supplementary material for: Association between visual acuity, lesion activity markers and retreatment decisions in neovascular age-related macular degeneration
Source: Eye (Lond). 2020 Feb 17;34(12):2249–56. doi: 10.1038/s41433-020-0799-y (PMC7784949; doi:10.1038/s41433-020-0799-y)
Supplement: Supplementary file 3 — Supplemental Table 2 [file 41433_2020_799_MOESM3_ESM.docx]

**Supplementary Table 2.** Association of VA with number of clinic visits with absence of IRF (a) or SRF (b) during the maintenance phase (i.e., Months 3–12) and comparisons with eyes with missing anatomical parameters (c).

**(a)**

| **Number of visits with IRF absent from Month 3 to Month 12** | **N** | **Age Mean (SD)** | **Number of injections in Year 1**  **(Baseline–Month 12; Mean [SD])** | **Number of injections in Year 1**  **(Month 3–Month 12; Mean [SD])** | **Loading phase completed (%)** | **Change in VA at Month 12 (Mean [SD])** |
| --- | --- | --- | --- | --- | --- | --- |
| **Missing** | 113 | 78.3 (7.0) | 5.2 (1.7) | 2.5 (1.6) | 77.0% | 2.4 (12.6) |
| **0 visits with IRF absent** | 19 | 81.3 (5.5) | 6.7 (1.2) | 3.7 (1.2) | 94.7% | -8.8 (18.3) |
| **1 visit with IRF absent** | 72 | 81.0 (5.6) | 5.8 (2.0) | 2.9 (2.0) | 94.4% | -0.4 (12.9) |
| **2 visits with IRF absent** | 45 | 80.0 (6.0) | 5.5 (2.0) | 2.5 (2.0) | 97.8% | 5.3 (12.6) |
| **3 visits with IRF absent** | 21 | 78.5 (7.2) | 5.8 (2.0) | 2.8 (2.0) | 95.2% | -0.3 (17.9) |
| **4 visits with IRF absent** | 11 | 80.8 (5.5) | 6.9 (1.1) | 3.9 (1.2) | 90.9% | 0.5 (8.9) |
| **5 visits with IRF absent** | 19 | 78.6 (6.0) | 6.4 (1.7) | 3.2 (1.8) | 100.0% | 5.4 (9.7) |
| **≥6 visits with IRF absent** | 21 | 79.6 (6.2) | 8.3 (2.0) | 5.2 (1.8) | 95.2% | 7 (11.3) |

**(b)**

| **Number of visits with SRF absent from Month 3 to Month 12** | **N** | **Age Mean (SD)** | **Number of injections in Year 1**  **(Baseline–Month 12; Mean [SD])** | **Number of injections in Year 1**  **(Month 3–Month 12; Mean [SD])** | **Loading phase completed (%)** | **Change in VA at month 12 (Mean [SD])** |
| --- | --- | --- | --- | --- | --- | --- |
| **Missing** | 113 | 78.3 (7.0) | 5.2 (1.7) | 2.5 (1.6) | 77.0% | 2.4 (12.6) |
| **0 visits with SRF absent** | 27 | 78.9 (6.3) | 6.9 (1.5) | 3.9 (1.5) | 100.0% | 1.6 (12.5) |
| **1 visit with SRF absent** | 82 | 80.1 (6.2) | 5.9 (1.9) | 2.9 (1.9) | 95.1% | -1.5 (15.2) |
| **2 visits with SRF absent** | 35 | 80.6 (5.3) | 5.5 (2.3) | 2.5 (2.3) | 91.4% | 4.4 (13.7) |
| **3 visits with SRF absent** | 19 | 80.9 (6.3) | 5.9 (2.2) | 2.8 (2.1) | 94.7% | -2.4 (14.3) |
| **4 visits with SRF absent** | 12 | 81.0 (6.1) | 7.4 (1.5) | 4.3 (1.3) | 100.0% | 1.0 (13.1) |
| **5 visits with SRF absent** | 17 | 78.5 (6.2) | 6.5 (2.2) | 3.4 (2.2) | 100.0% | 5.9 (8.6) |
| **≥6 visits with SRF absent** | 16 | 82.1 (4.6) | 7.4 (1.8) | 4.5 (1.7) | 93.8% | 9.3 (11.2) |

**(c)**

|  |  | **IRF absent** | | **SRF absent** | |
| --- | --- | --- | --- | --- | --- |
|  | **Eyes with missing anatomical parameters** | **<2 visits** | **≥2 visits** | **<2 visits** | **≥2 visits** |
| **N (Eyes)** | 113 | 91 | 117 | 109 | 99 |
| **Age, Mean (SD)** | 78.3 (7.0) | 81.1 (5.6) | 79.5 (6.2) | 79.8 (6.2) | 80.6 (5.7) |
| **Number of injections from Month 3-12,  Mean (SD)** | 2.5 (1.6) | 3.0 (1.9) | 3.3 (2.1) | 3.1 (1.8) | 3.25 (2.2) |
| **Eyes with completed loading phase, %** | 77.0% | 94.5% | 96.6% | 96.3% | 95.0% |
| **VA at baseline, Mean (SD)** | 54.8 (8.0) | 56.3 (8.6) | 53.4 (9.1) | 56.0 (9.1) | 53.1 (8.6) |
| **VA change from baseline to month 12, Mean (SD)** | 2.4 (12.6) | -2.1 (14.5) | 4.2 (12.9) | -0.72 (14.6) | 3.8 (12.9) |
| **VA change from baseline to month 12, Median (IQR)** | 3 (-5, 12) | 2 (-8, 7) | 5 (-3, 14) | 2 (-6, 9) | 5 (-3, 12) |
|  |  | p-value=0.006* | | p-value=0.042* | |

*Mann-Whitney test of median values. ETDRS, Early Treatment Diabetic Retinopathy Study; IQR, interquartile range; IRF, intraretinal fluid; SD, standard deviation; SRF, subretinal fluid; VA, visual acuity.
