# Supplementary material for: Association between visual acuity, lesion activity markers and retreatment decisions in neovascular age-related macular degeneration
Source: Eye (Lond). 2020 Feb 17;34(12):2249–56. doi: 10.1038/s41433-020-0799-y (PMC7784949; doi:10.1038/s41433-020-0799-y)
Supplement: Supplementary file 4 — Supplemental Table 3 [file 41433_2020_799_MOESM4_ESM.docx]

**Supplementary Table 3.** Sensitivity analysis of the association of VA with number of clinic visits with absence of IRF or SRF during the maintenance phase (i.e., Months 3–12).

|  | **IRF absent** | | **SRF absent** | | |
| --- | --- | --- | --- | --- | --- |
|  | **<2 visits** | **≥2 visits** | **<2 visits** | **≥2 visits** | |
| **N (eyes)** | 201 | 120 | 219 | 102 | |
| **Age, Mean (SD)** | 79.5 (6.5) | 79.5 (6.2) | 79.0 (6.6) | 80.6 (5.8) | |
| **Number of injections from Month 3-12,  Mean (SD)** | 2.8 (1.71) | 3.2 (2.1) | 2.8 (1.7) | 3.2 (2.2) | |
| **Eyes with completed loading phase, %** | 84.6% | 96.7% | 86.3% | 95.1% | |
| **VA at baseline, Mean (SD)** | 55.5 (8.4) | 53.4 (9.0) | 55.4 (8.6) | 53.1 (8.5) | |
| **VA change from baseline to Month 12, Mean (SD)** | 0.3 (13.7) | 4.1 (12.9) | 0.8 (13.7) | 3.7 (12.9) | |
| **VA change from baseline to Month 12, Median (IQR)** | 3 (-5, 9) | 5 (-3, 14) | 3 (-5, 10) | 5 (-3, 12) | |
|  | p-value = 0.036* | | p-value = 0.111* | |  |

*Mann-Whitney test. ETDRS, Early Treatment Diabetic Retinopathy Study; IQR, interquartile range; IRF, intraretinal fluid; SD, standard deviation; SRF, subretinal fluid; VA, visual acuity.
